# Gender identification of the horsehair crab, *Erimacrus isenbeckii* (Brandt, 1848), by image recognition with a deep neural network

**DOI:** 10.1038/s41598-023-46606-x

**Published:** 2023-11-13

**Authors:** Yoshitaka Ueki, Kenji Toyota, Tsuyoshi Ohira, Ken Takeuchi, Shin-ichi Satake

**Affiliations:** 1 Department of Applied Electronics, Faculty of Advanced Engineering, Tokyo University of Science, 6‑3‑1 Niijuku, Katsushika‑ku, Tokyo 125‑8585 Japan; 2 Noto Marine Laboratory, Institute of Nature and Environmental Technology, Kanazawa University, Ogi, Noto‑cho, Ishikawa 927‑0553 Japan; 3 Department of Biological Science and Technology, Faculty of Advanced Engineering, Tokyo University of Science, 6‑3‑1 Niijuku, Katsushika‑ku, Tokyo 125‑8585 Japan; 4 Department of Science, Faculty of Science, Kanagawa University, 3-27-1 Rokkakubashi, Kanagawa-ku, Yokohama-shi, Kanagawa 221‑8686 Japan; 5 Oshamambe Division, Institute of Arts and Sciences, Tokyo University of Science, 102-1 Tomino, Oshamambe-cho, Yamakoshi-gun, Hokkaido 049-3514 Japan

**Keywords:** Machine learning, Marine biology

## Abstract

Appearance-based gender identification of the horsehair crab [*Erimacrus isenbeckii* (Brandt, 1848)] is important for preventing indiscriminate fishing of female crabs. Although their gender is easily identified by visual observation of their abdomen because of a difference in the forms of their sex organs, most of the crabs settle with their shell side upward when placed on a floor, making visual gender identification difficult. Our objective is to use deep learning to identify the gender of the horsehair crab on the basis of images of their shell and abdomen sides. Deep learning was applied to a photograph of 60 males and 60 females captured in Funka Bay, Southern Hokkaido, Japan. The deep learning algorithms used the AlexNet, VGG-16, and ResNet-50 convolutional neural networks. The VGG-16 network achieved high accuracy. Heatmaps were enhanced near the forms of the sex organs in the abdomen side (F-1 measure: 98%). The bottom of the shell was enhanced in the heatmap of a male; by contrast, the upper part of the shell was enhanced in the heatmap of a female (F-1 measure: 95%). The image recognition of the shell side based on a deep learning algorithm enabled more precise gender identification than could be achieved by human-eye inspection.

## Introduction

The horsehair crab [*Erimacrus isenbeckii* (Brandt, 1848)] is a high-quality marine product in Japan. The capture of female horsehair crabs is prohibited in Hokkaido, Japan, and their capture for academic research purposes requires permission. Immediate gender identification and selective capture within a limited time and in the limited space on a fishing boat are important. Fishermen in Hokkaido, who have many years of experience in the fisheries industry, can distinguish crab genders by visual inspection. Some academic research regarding the gender identification of crabs has been reported. Toyota et al. investigated morphometric gender identification of the horsehair crab^1^. Gender identification based on 3D measurement of the shell geometry of the horsehair crab has also been investigated^2^.

Computer vision can automatically detect the characteristics of medical images^3,4^ on the basis of intuitive and high-precision characteristics at high speed and is becoming an important method in medical fields. Studies in the field of medical gender identification have used, for example, an eyeground image^5^ and an image of a bone^6,7^. In biology, mosquito classification has been studied^8–11^. However, the literature contains few studies^12–14^ on the recognition and classification of crabs using machine learning and deep learning methods. Among the limited examples, Wu et al.^15^ used abdomen images for swimming crabs and mud crabs to identify individual crabs via deep neural networks.

Zhang et al. have suggested a shell detection–recognition method that combines principal component analysis (PCA) with YOLOv5 (You Only Look Once v5), resulting in an improved method to recognize individual Chinese mitten crabs^16^. Cui et al.^17^ developed a gender classification method for the Chinese mitten crab using a deep convolutional neural network. Their original algorithm comprised a batch normalization technique and a dropout technique, and their proposed method achieved 98.90% classification accuracy. Notably, gender identification of the horsehair crab using machine learning and deep learning has not been reported.

In the present study, we investigated gender identification and its prediction precision in the shell and abdomen images of horsehair crabs using deep learning. For deep learning, we employed established and conventional network architectures. In addition, visualization of class-discriminative localization maps of the present deep learning models explains which parts of the horsehair crab images are focused on in the process of gender identification.

## Methods

### Horsehair crab dataset

A total of 120 crabs consisting of 60 males and 60 females were collected in Funka Bay, Pacific Ocean, Southern Hokkaido, Japan, in May 2023. Images of the shell and abdomen geometry of the horsehair crabs were used in the present study. Fishing permission for horsehair crabs used in this study was granted by the Hokkaido Governor. The images were taken using a camera (ILCE-6600, SONY) with a macro lens (SEL30M35, SONY). Each original image had a resolution of 6000 × 4000 pixels with 24-bit RGB channels. The original images were cropped to smaller sizes of 3400 × 3400 pixels to remove extra edges of the images. The cropped images were then compressed to 224 × 224 pixels to match the input sizes of the following deep convolution neural network (DCNN) models. Through this manual image acquisition process, we collected ~ 120 images for each target crab for the shell or abdomen geometry, totaling ~ 240 images.

### Sec2

**Figure 1:**
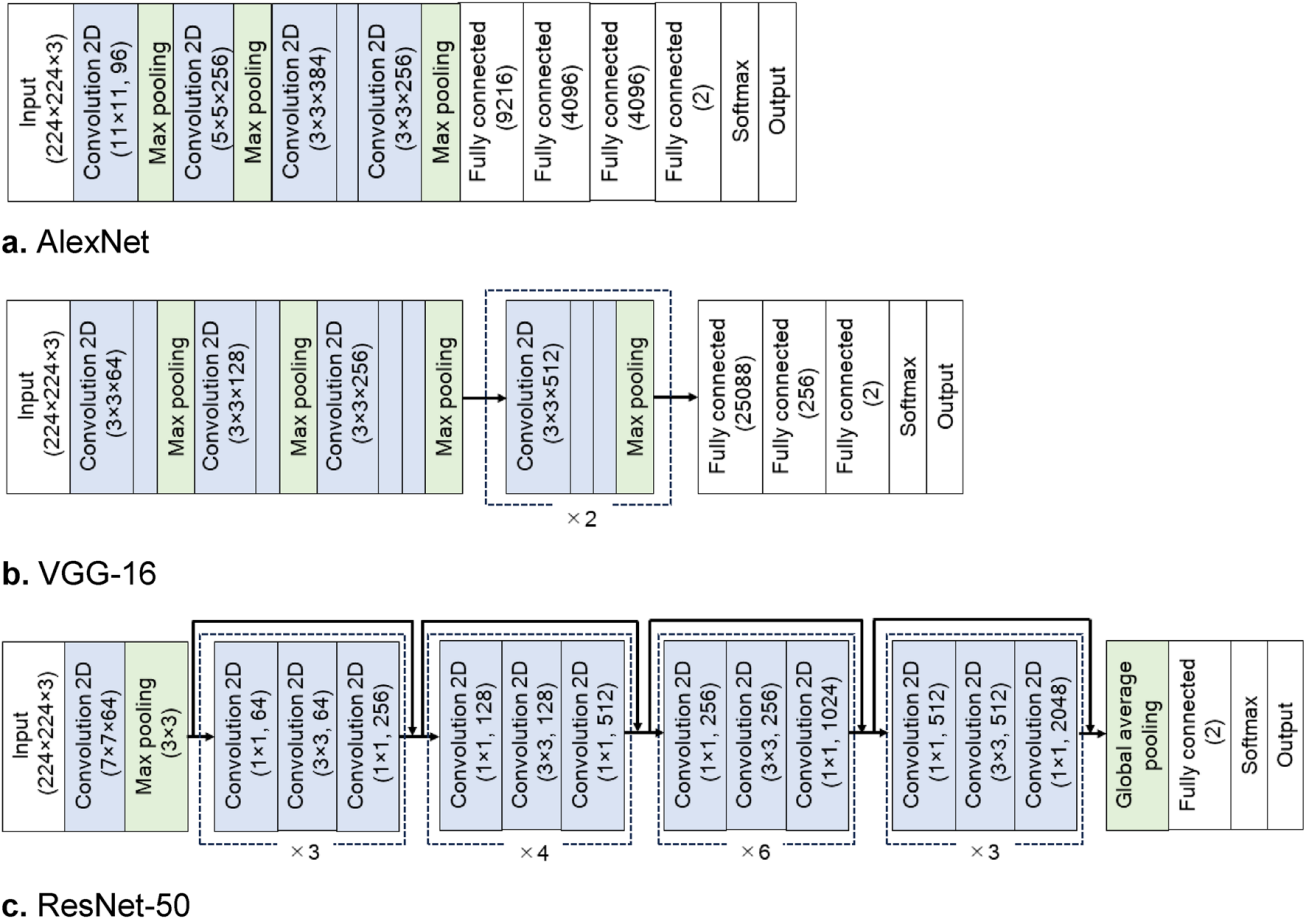
Deep convolutional neural network architectures used in the present study: (**a**) AlexNet, (**b**) VGG-16, and (**c**) ResNet-50.

In the present study, we used the following established DCNN models: AlexNet^18^, VGG-16^19^, and ResNet-50^20^. These DCNN architectures are illustrated in Fig. 1. They consist of feature extraction and classification parts. The feature extraction part is formed by a series of convolution layers. The input RGB images are 224 × 224 pixels. Through successive convolution layers, the spatial dimensions of the feature maps were reduced from 224 × 224 to 13 × 13 (AlexNet), 14 × 14 (VGG-16), and 7 × 7 (ResNet-50). At the ends of the feature extraction part, the feature maps were flattened into a one-dimensional array for the classification through downstream fully connected (FC) layers. The present DCNN models were designed to classify binary classes of female and male so that the classification part was modified from its original structure. Outputs of the final FC layer for a target class *c*, denoted by *y*^*c*^, were fed into the softmax function to obtain the classification probability of each target class.

### Training the DCNNs

**Figure 2:**
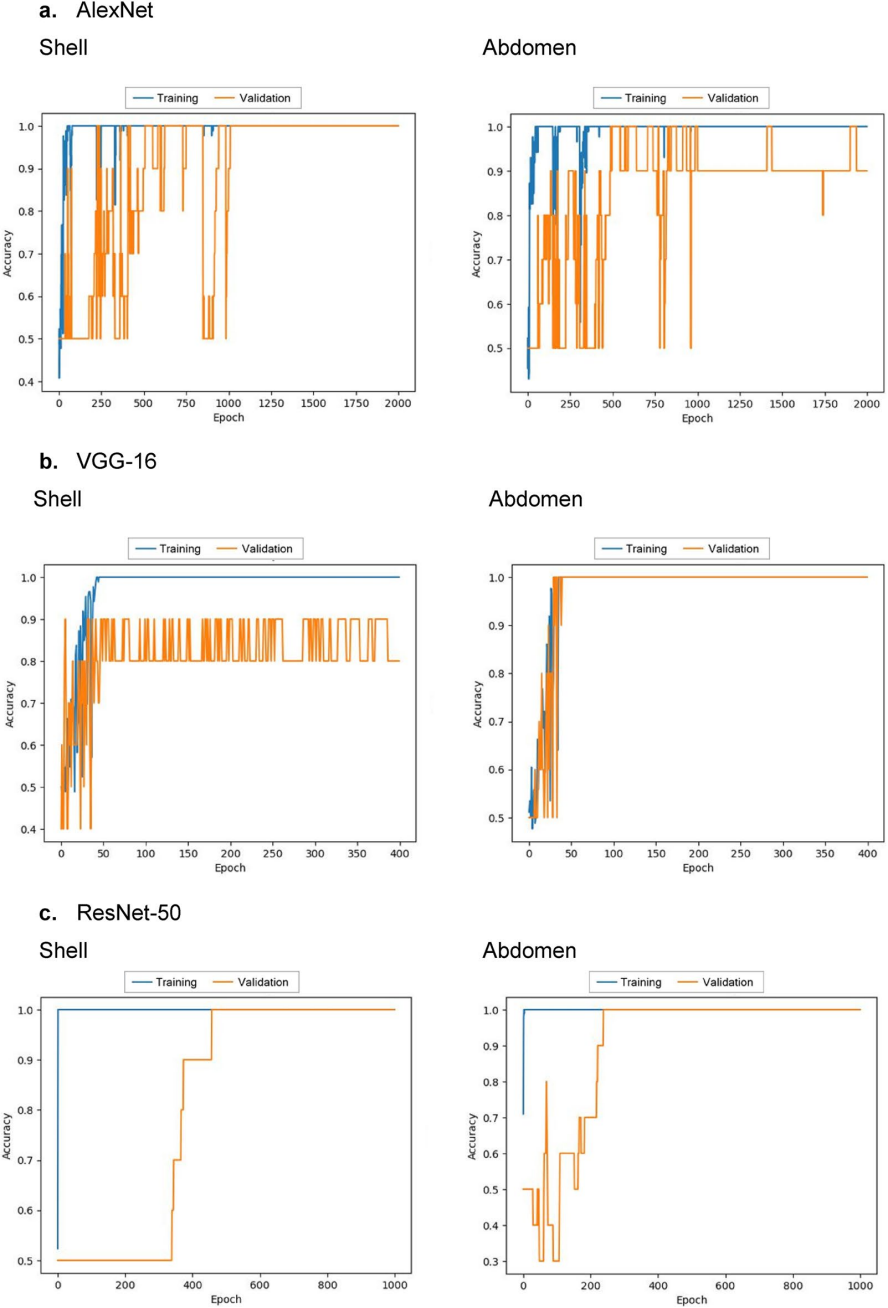
Validation accuracy of the models during the training: (**a**) AlexNet, (**b**) VGG-16, and (**c**) ResNet-50. In all the models, optimal validation accuracy is achieved when fine-tuning is applied for VGG-16 and ResNet-50.

For the present dataset of the crab images, we employed fine-tuning to train the aforementioned DCNN models, except AlexNet. Pre-trained weights of AlexNet were not available. The model weights were initialized by the uniform random distribution and trained from scratch. In the cases of VGG-16 and ResNet-50, model weights pre-trained on ImageNet were used for their initial parameters. In the case of VGG-16, the pre-trained weights were partially retrained by the present dataset of the crab images. Only the last block consisted of the three consecutive convolution layers and the single max pooling layer before the FC layers were retrained together with the FC layers. In the case of ResNet-50, the pre-trained weights were totally retrained. As an optimizer, the stochastic gradient descent (SGD) was used with the learning rate set to 0.01. The categorial cross-entropy was employed as a loss function. The *K*-fold cross-validation was performed (*K* = 5 in the present study). The whole dataset was randomly partitioned into 80–20%, where 80% of the whole dataset was used for the training. Ten percent of the training dataset was employed for the validation during the training to monitor the training progress (Fig. 2). The remaining 20% of the whole dataset was reserved for testing of the classification performance. Each partitioned dataset contained the same quantities of female and male data. The training epoch was set to 2000, 400, and 1000 in the cases of AlexNet, VGG-16, and ResNet-50, respectively. Depending on the network architectures, the epoch to reach the optimal validation accuracy differed. For the model training and testing, we used the Keras framework on a Python 3.9.16 environment and an NVIDIA GeForce RTX 4080 16 GB GPU platform. Keras 2.9.0, TensorFlow 2.9.1, and NumPy 1.22.3 were employed.

## Results and discussion

### Classification performance

In the present study, we evaluated both the F-1 measure and the accuracy, which are defined as

$${\text{Accuracy}} = \frac{TP + TN}{{TP + TN + FP + FN}}$$

$${\text{F - 1}} {\text{measure}} = \frac{2TP}{{2TP + FP + FN}}$$

where *TP*, *TN*, *FP*, and *FN* represent true-positive, true-negative, false-positive, and false-negative results in the binary classification, respectively. Table 1 summarizes the classification performance of the present DCNN models. The F-1 measure is the five-time averaged value with the corresponding standard deviation. Notably, all the DCNN models achieved significantly high F-1 measures with a relatively low standard deviation, indicating that they all demonstrated sufficiently accurate and stable classification capability to distinguish crab gender. Although AlexNet has a relatively shallow network architecture, it demonstrated a relatively high classification capability, where the F-1 measure was greater than approximately 90% in both the shell and abdomen cases. VGG-16 and ResNet-50, which have deeper network architectures, achieved slightly higher classification performance. We speculatively attribute this better performance to the better feature extraction of the deeper network architecture and to the fine-tuning achieving more optimal weights for higher classification performance.

**Table 1:** Summary of the classification performance investigated in the present work.

DCNN model	Target images	F-1 measure	Standard deviation
AlexNet	Shell	0.921	0.0374
	Abdomen	0.946	0.0474
VGG-16	Shell	0.948	0.0329
	Abdomen	0.983	0.0205
ResNet-50	Shell	0.954	0.0522
	Abdomen	1.00	0.00

The high precision led to the results discussed in the preceding paragraph for VGG-16. To verify the result, an explainable visualization method for gender identification is presented in the following subsection.

### Visualization of feature activation

**Figure 3:**
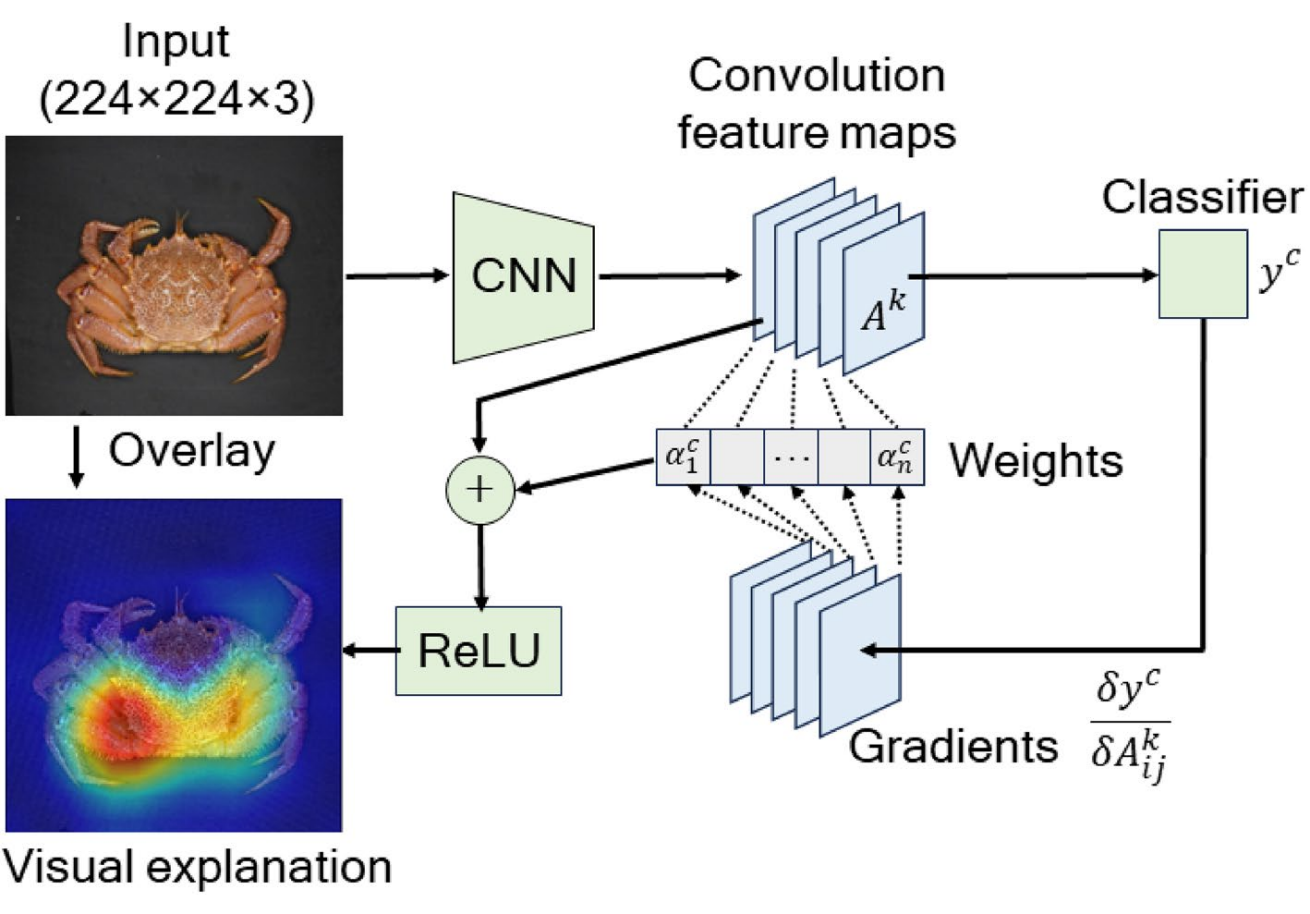
Schematic of the gender identification and visual explanation in the DCNN model. The class of a given horsehair crab image is predicted by two steps: (1) extracting hierarchical features and (2) classifying these features. In the feature-extraction step, feature maps are generated by filters at each convolution layer. The feature maps are used for the visual explanation by Grad-CAM.

**Figure 4:**
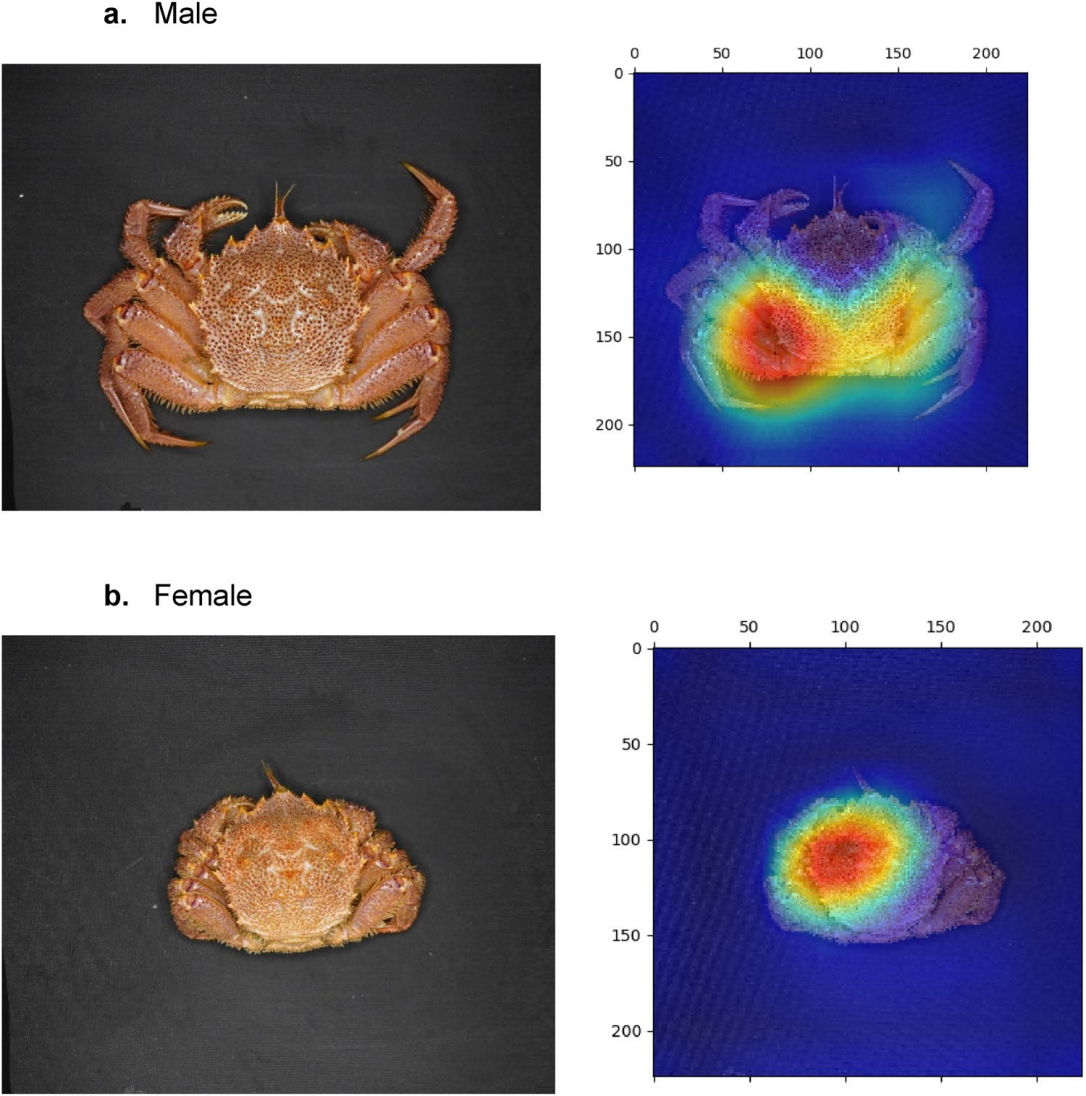
Two major cases of classification in the shell side. Samples are shown for a (**a**) male and (**b**) female with their discriminative regions as heatmaps.

**Figure 5:**
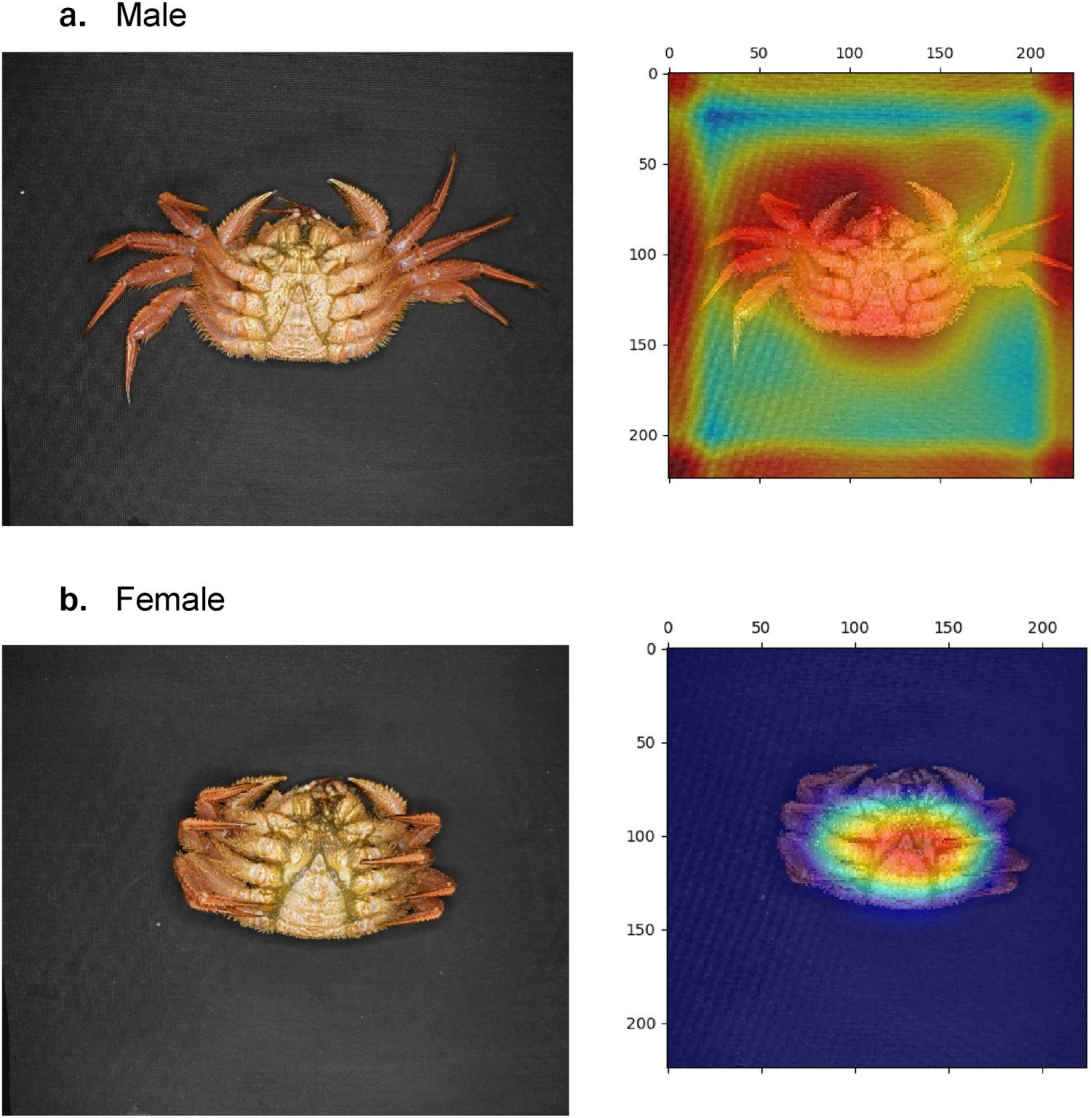
Two major cases of classification in the abdomen side. Samples are shown for a (**a**) male and (**b**) female with their discriminative regions as heatmaps.

To further comprehend the gender identification by the DCNN models, we employed Grad-CAM^21^ to visually explain the DCNN classification results. The class-discriminative localization map *L*^*c*^ of width *u* and height *v* for class *c* was computed using the following equation:

$$L^{c} = {\text{ReLU}}\left( {\mathop \sum \limits_{k}^{n} \alpha_{k}^{c} A^{k} } \right)$$

where ${\alpha }_{k}^{c}$ denotes the neuron importance weights and *A*^*k*^ denotes feature maps of a convolution layer. The neuron importance weights were evaluated by global average pooling the gradients as follows:

$$\alpha_{k}^{c} = \frac{1}{uv}\mathop \sum \limits_{i}^{u} \mathop \sum \limits_{j}^{v} \frac{{\partial y^{c} }}{{\partial A_{ij}^{k} }}$$

where $\frac{\partial {y}^{c}}{\partial {A}_{ij}^{k}}$ denotes the gradients via backpropagation until the convolution layer. Figure 3 shows the Grad-CAM scheme. The class-discriminative localization maps were originally coarse. They were resized to match the size of the input images by bilinear interpolation and then overlayed on the input image. Figures 4 and 5 show typical visualization explanations obtained by the Grad-CAM scheme for the VGG-16 neural network. The class-discriminative localization maps were generated from the last convolution layer in the VGG-16 architecture.

Figure 4 shows the analysis of the image of the shell side. The heatmap is enhanced in the lower area for a male and in the upper area for a female. The highest point of these contours is completely divided at the carapace width (CW). Because the upper half of the female’s shell is highlighted and the lower half of the male’s shell is highlighted, in the case of gender identification of this shell side, using images of the whole shell is inevitable, consistent with Toyota et al.^1^ morphologic gender identification of the shell.

The abdomen geometry of a horsehair crab is shown in Fig. 5. Although Wu et al.^15^ identified individual crabs using a part-based deep learning network for texture features of the abdomen, our approach achieved gender identification without such a partition strategy. For this reason, in the case of gender identification, the partition strategy is not required for the present classification scheme because the form of the sexual organs is geometrically clear.

## Conclusions

We demonstrated the effectiveness of deep neural networks for image recognition and revealed gender differences in the shell and abdomen geometry of the horsehair crab. We created a dataset that contained ~ 120 images of each shell and abdomen geometry of the horsehair crab. From the images of the abdomen geometry of a crab, the model could distinguish between a male and a female in the form of the sex organs, similar to gender identification by human visual inspection. The analysis of the images of the shell side included a more interesting result: Even though gender classification was impossible by human visual inspection, the present deep learning models enabled male and female classification with high precision. The F-1 measure reached approximately 95%. The discriminative region in the visual explanation was concentrated on the upper side of the shell for females and on the lower side of the shell for males.

## Data Availability

The datasets used and/or analyzed during the current study may be made available from the corresponding author and the first author (e-mail: ueki@rs.tus.ac.jp) on reasonable request.

## References

[1] Toyota K, Izumi K, Ichikawa T, Ohira T, Takeuchi K (2020). Morphometric approaches reveal sexual differences in the carapace shape of the horsehair crab, *Erimacrus isenbeckii* (Brandt, 1848). Aquat. Anim..

[2] Toyota K, Arai Y, Miyagawa S, Kogo Y, Takeuchi K (2021). Novel validating indices to indicate sexual differences in the horsehair crab *Erimacrus isenbeckii* (Brandt, 1848). Aquat. Anim..

[3] Ker J, Wang LP, Rao J, Lim T (2018). Deep learning applications in medical image analysis. IEEE Access.

[4] Babenko B (2022). Detection of signs of disease in external photographs of the eyes via deep learning. Nat. Biomed. Eng..

[5] Korot E (2021). Predicting sex from retinal fundus photographs using automated deep learning. Sci. Rep..

[6] Intasuwan P, Palee P, Sinthubua A, Mahakkanukrauh P (2022). Comparison of sex determination using three methods applied to the greater sciatic notch of os coxae in a Thai population: Dry bone morphology, 2-dimensional photograph morphometry, and deep learning artificial neural network. Med. Sci. Law.

[7] Malatong Y, Intasuwan P, Palee P, Sinthubua A, Mahakkanukrauh P (2023). Deep learning and morphometric approach for sex determination of the lumbar vertebrae in a Thai population. Med. Sci. Law.

[8] Minakshi M, Bharti P, Bhuiyan T, Kariev S, Chellappan S (2020). A framework based on deep neural networks to extract anatomy of mosquitoes from images. Sci. Rep..

[9] Kittichai V (2021). Deep learning approaches for challenging species and gender identification of mosquito vectors. Sci. Rep..

[10] Park J, Kim DI, Choi B, Kang W, Kwon HW (2020). Classification and morphological analysis of vector mosquitoes using deep convolutional neural networks. Sci. Rep..

[11] Pataki BA (2021). Deep learning identification for citizen science surveillance of tiger mosquitoes. Sci. Rep..

[12] Wang DY, Vinson R, Holmes M, Seibel G, Tao Y (2018). Convolutional neural network guided blue crab knuckle detection for autonomous crab meat picking machine. Opt. Eng..

[13] Wang, D., Holmes, M., Vinson, R., Seibel, G. & Tao, Y. in *2018 ASABE Annual International Meeting ASABE Paper No. 1800570* 1 (ASABE, St. Joseph, 2018).

[14] Wang H (2023). Quality grading of river crabs based on machine vision and GA-BPNN. Sensors.

[15] Wu CJ (2023). A part-based deep learning network for identifying individual crabs using abdomen images. Front. Mar. Sci..

[16] Zhang JZ, Wang SX, Zhang SM, Li JK, Sun YY (2023). Research on target detection and recognition algorithm of *Eriocheir sinensis* carapace. Multimed. Tools Appl..

[17] Cui YH, Pan TH, Chen S, Zou XB (2020). A gender classification method for Chinese mitten crab using deep convolutional neural network. Multimed. Tools Appl..

[18] Krizhevsky A, Sutskever I, Hinton GE (2017). ImageNet classification with deep convolutional neural networks. Commun. ACM.

[19] Simonyan, K. Z., A. in *ICLR* (San Diego, 2015).

[20] Shafiq M, Gu ZQ (2022). Deep residual learning for image recognition: A survey. Appl Sci.

[21] Selvaraju RR (2020). Grad-CAM: Visual explanations from deep networks via gradient-based localization. Int. J. Comput. Vis..

